# Anticholinergic Drugs in Nonallergic Rhinitis

**DOI:** 10.1097/WOX.0b013e3181b35336

**Published:** 2009-08-15

**Authors:** Robert Naclerio

**Affiliations:** 1 Section of Otolaryngology--Head and Neck Surgery, The University of Chicago, Chicago, IL

**Keywords:** anticholinergic, atropine, ipratropium bromide, rhinitis, nonallergic rhinitis

## Abstract

**Background:**

The parasympathetic nervous system contributes to the pathophysiology of multiple forms of allergic and nonallergic rhinitis. Stimulation of the parasympathetic nervous system leads to glandular activation, which produces watery secretions. In excess, these secretions discharge from the anterior Nares and produce the symptom of watery anterior rhinorrhea.

**Method:**

Review of literature.

**Results:**

Treatment with topical, intranasal anticholinergic drugs inhibits activation of the nasal mucosal glands and is effective in reducing the watery secretions associated with parasympathetic stimulation of the glands with little, if any, effect on the symptoms of congestion and sneezing. In general, these drugs have no systemic adverse effects, but can cause crusting and local irritation.

**Conclusion:**

Anticholinergic drugs are useful for the treatment of anterior rhinorrhea associated with allergic and nonallergic rhinitis.

## Introduction

The nervous system plays an important role in nasal physiology and pathophysiology by functioning to provide rapid responses to physical and chemical stimuli. The sensory, parasympathetic, and sympathetic systems all contribute to these responses. Up-regulation of this system can occur at any level and is referred to as neural hyperresponsiveness.

Central neuronal reflexes involving the parasympathetic nervous system are the best studied component of the nasal nervous system and contribute to multiple forms of rhinitis. Stimulation of the trigeminal nerve sends inputs to the midbrain, where parasympathetic impulses originate, and subsequently move to the nose via the preganglionic fibers (superior salivary nucleus, CN VII through facial genu, greater petrosal, vidian), where they synapse in the pterygopalatine ganglion. Postganglionic nerves travel with branches of the nasopalatine nerve to the minor salivary glands in the nose.

Acetylcholine is released from the postganglionic nerves and stimulates muscarinic receptors on the glands. There are 5 muscarinic receptor subtypes, labeled M1 to M5. M1, M2, and M3 receptors are found on glands, arteries, veins, and epithelium. M4 receptors are found on arteries, and M5 receptors are found on glands and arteries. M3 is the most common receptor subtype [1]. M1, M3, and M5 couple to inositol polyphosphate, whereas M2 and M4 inhibit the generation of cAMP [2]. In addition to secreting acetylcholine, postganglionic fibers release vasoactive intestinal peptide, peptide histidine methionine, and peptide histidine valine. Postganglionic nerve bodies also contain nitric oxide synthase. These latter substances may be responsible for atropine-resistant vasodilatation associated with parasympathetic stimulation.

Anticholinergic agents block the binding of acetylcholine to the muscarinic receptors. The drugs can be delivered topically or systemically. Methacholine challenges of the nasal mucosa, which stimulates the gland directly via muscarinic receptors, have been used to show the effectiveness and duration of the action of anticholinergic drugs [3,4].

## Role of Parasympathetic Glandular System in Nasal Physiology

A major function of the nose is to warm and humidify inspired air. The role of glandular secretion in this process and subsequently the effect of blocking glandular stimulation are not fully understood.

In a study by Ingelstedt and Ivstan, the effect of atropine on humidification of air in the nose was studied by psychrometer [5]. They showed that a subcutaneous injection of 1 mg atropine greatly impaired the humidifying capacity of the nose in healthy subjects. The authors concluded that atropine-inhibitable glandular secretions provide the major source of water for humidification. A criticism of this study was that air was blown from the nasopharynx and measured as it exited the nostril, the direction being the reverse of that by which air is normally humidified. Kumlien and Drettner [6] measured nasal conditioning in 16 healthy volunteers on 2 separate visits, the first visit without medication and the other visit after application of 0.06 mg of ipratropium bromide into one nostril. There were no significant differences in nasal conditioning between the 2 visits. Consistent with Kumlien and Drettner's finding, we showed no significant differences in nasal conditioning after administration of ipratropium bromide at a flow rate of 5 liter/min [7] and normal saline. Our studies, however, showed that with the use of ipratropium bromide at flow rates of 10 and 20 liters/min, such as occur with mild to moderate exercise, led to more water evaporation and conditioning. Thus, we have shown that ipratropium bromide, which is effective for the treatment of rhinorrhea, increased the ability of the nose to condition air. This effect is probably related to an increase in superficial nasal mucosal blood flow rather than to changes in nasal volume. Our results clearly indicate that blocking of the nasal glands does not impair the ability of the nose to warm and humidify air, suggesting that there are other sources providing fluid for the humidification process and emphasizing the complex responses of a physiologic system like the nose that cannot be predicted a priori. The information that ipratropium bromide does not impair nasal conditioning is useful clinically in the treatment of patients who have rhinorrhea and asthma, because this agent leads to better-conditioned air and thus does not adversely affect the lower airway.

## Anticholinergic Drugs in Rhinitis

The involvement of the parasympathetic nervous system has been shown to have an effect on multiple nasal diseases. Georgitis showed that atropine sulfate, a nonselective muscarinic receptor antagonist, improved severe rhinorrhea in patients with perennial allergic rhinitis, whereas the other nasal symptoms were not improved significantly [8]. Georgitis reported similar results for treatment with ipratropium, a nonselective muscarinic antagonist, in patients with perennial allergic rhinitis [9]. Kaiser and colleagues studied 176 patients who had perennial allergic rhinitis and found that 42 and 84 *μ*g/nostril of ipratropium bromide 3 times a day were equally effective in reducing the severity and duration of rhinorrhea compared with placebo, while resulting in no significant effect on sneezing or congestion [10]. Mygind and Borum showed in a double-blind, placebo-controlled, cross-over trial in 20 subjects who had severe chronic perennial rhinitis with watery discharge as the dominant symptom that ipratropium, 80 *μ*g 4 times a day, reduced rhinorrhea with no effect on blockage or sneezing [11]. Bonadonna and colleagues showed the effectiveness of 80 *μ*g 2 times a day of ipratropium nasal spray on cold-induced rhinorrhea and tissue weights in skiers with and without allergies [12]. Silvers showed the effect of a single dose of 0.005% solution of atropine sulfate on the rhinorrhea induced by skiing [13]. Ostberg and colleagues showed that 400 *μ*g 4 times a day of ipratropium bromide reduced nose blowing by 56% and the amount of blown secretions collected on tissues by 58% compared with placebo in subjects who had a common cold, suggesting that nearly 60% of the discharge in the first days of a cold are reflex-mediated [14]. Similar findings were obtained by Hayden and colleagues [15]. Ipratropium bromide nasal 0.06% was shown to have beneficial effects on all nasal symptoms in an open study of children 2 to 5 years old who had rhinorrhea secondary to a common cold or allergy [16].

Anticholinergics have been combined with other topical agents in the treatment of perennial rhinitis. Dockhorn and colleagues reported on the use of 42 *μ*g 2 times a day of ipratropium bromide nasal spray 0.03% combined with beclomethasone 84 *μ*g per nostril 2 times a day versus either agent alone [17]. Mixed matching placebos were used. After a run-in period, each agent was compared with placebo; then after 4 weeks, they evaluated the combination of both therapies. Five hundred thirty-three patients, 279 allergic 274 nonallergic, ages 8 to 75 years were included. To enter, each subject had to have mild rhinorrhea 2 hours per day for a week plus mild congestion or sneezing. A negative Water's radiograph was required. Ipratropium alone had a faster onset of action. Beclomethasone was more effective at reducing congestion and sneezing. The investigators found that the combination was more effective than either agent alone or vehicle in reducing rhinorrhea. Ipratropium was more effective than placebo in previous nonsteroid responders. Unfortunately, the results for the subgroups of allergic and nonallergic rhinitis subjects were not shown.

Eccles and colleagues showed that the combination of ipratropium and xylometazoline was effective for the treatment of upper respiratory tract infections [18]. Ipratropium reduced the rhinorrhea, whereas xylometazoline blocked the congestion.

Finn and colleagues [19] showed that terfenadine, 60 mg 2 times a day, plus ipratropium nasal spray 0.03% (42 *μ*g) 3 times a day was more effective than terfenadine plus vehicle in the control of the severity and duration of rhinorrhea in subjects with perennial rhinitis (n = 91 nonallergic rhinitis; n = 114 perennial allergic rhinitis; results not broken down by group). First-generation antihistamines have anticholinergic activity. However, there are no clinical data showing their effectiveness in vasomotor rhinitis [20].

## Challenge Studies

Cruz showed the effect of atropine on cold, dry air challenge of the upper airway [21]. In support of the lack of effect of anticholinergic agents on congestion, Shusterman and colleagues studied the response to chlorine gas [22]. In allergic subjects, chlorine gas, an irritant, caused congestion, more so in allergic individuals than in controls, but this was unaffected by treatment with ipratropium bromide, 0.06%. In contrast to most studies, McLean and colleagues showed that ammonia increased nasal airway resistance in nonallergic rhinitic subjects and that atropine inhibited the response [23]. Most other studies suggest that anticholinergics have little or no effect on congestion and its surrogate markers.

Stjarne and colleagues showed that both capsaicin and nicotine stimulation led to reflex cholinergic activity, which was blocked by atropine [24]. They found that the patients with vasomotor rhinitis who had rhinorrhea as a predominant symptom were more responsive, suggesting that vasomotor rhinitis patients could be divided into 2 groups based on their symptoms.

Reflex activation of nasal glands and hence rhinorrhea can be initiated outside the nasal cavity, an example being gustatory rhinitis [25]. Pretreatment with atropine significantly blocked rhinorrhea and secretions induced by a positive food-induced challenge. Likewise, reflexes initiated in the nose can affect the eyes and the bronchial tree [26].

## Adverse Effects

The most frequent nasal adverse events of ipratropium bromide were transient nasal dryness or epistaxis. No evidence of rebound was reported after discontinuing usage of the drug [27].

Less than 20% of a single dose of ipratropium bromide is absorbed from the nasal mucosa. Rare cases of systemic anticholinergic effects, such as dry mouth, ocular irritation, and blurred vision, have been reported [27].

## Other Treatments Affecting the Parasympathetic Nervous System

Botulinum toxin type A inhibits the release of acetylcholine from cholinergic nerve endings. Kim and colleagues performed a double-blind, placebo-controlled study in which they administered 2 units of botulinum toxin A into both inferior and middle turbinates of patients who had intrinsic rhinitis [28]. This produced a significant reduction in rhinorrhea (24-42%) and a 54% reduction in tissue usage, with no effect on sneezing or nasal stuffiness. The positive effect lasted 4 weeks. Intrinsic rhinitis was defined by history, negative skin or RAST tests, negative history of asthma or dermatitides, and no satisfactory response to treatment with an antihistamine or topical nasal steroid.

Besides blocking the central parasympathetic reflex after the release of acetylcholine, investigators have used capsaicin to deplete c-fibers of neuropeptides, reducing both the initiation of the central reflex and nasal hyperreactivity. Desensitization with capsaicin led to clinical improvement in patients who had vasomotor rhinitis and rhinorrhea [29]. Others have used silver nitrate or lasers to affect surface secretions [30,31].

Vidian neurectomy has also been used; however, side effects of dry eyes and recurrence of symptoms possibly related to reinnervation can occur, making this a rarely performed procedure [32-34]. This technique blocks more than the effects of acetylcholine and has effects on neuropeptide release and axonal reflexes.

## Conclusions

Anticholinergic drugs are effective in blocking parasympathetic-induced release of acetylcholine, which plays a role in the symptom of watery rhinorrhea in many forms of rhinitis. Ipratropium bromide is approved for the treatment of rhinorrhea associated with allergic and nonallergic rhinitis. Criteria for selecting responders are based primarily on symptom assessment. Knowing the response to prior treatments helps choose responders to ipratropium bromide.

## End Note

Dr. Naclerio discloses that he has received grant/research support from GlaxoSmithKline (GSK), Schering Plough Corporation, and Merck. He also is a consultant, or on an advisory board or the speakers bureau for those companies. Funding for the research described in this paper included a grant from the McHugh Otolaryngology Research Fund.

Presented at a roundtable conference held in December 2008 in Washington, DC. The meeting was sponsored by the TREAT Foundation (Washington, DC) and supported through an unrestricted educational grant from Meda Pharmaceuticals. The funding company did not have any input into the development of the meeting or the series, and the company was not represented at the roundtable meeting.
